# Influence of cross-sectional aspect ratio on biochar segregation in a bubbling fluidized bed

**DOI:** 10.1038/s41598-022-14282-y

**Published:** 2022-06-22

**Authors:** Hoon Chae Park, Hang Seok Choi

**Affiliations:** 1 Engineering and Construction Group, Samsung C&T, Seoul, 05288 Republic of Korea; 2 Department of Environmental Engineering, Yonsei University, Wonju, 26493 Republic of Korea

**Keywords:** Chemical engineering, Biofuels

## Abstract

In this study, computational particle fluid dynamics was applied to investigate the segregation characteristics of biochar in a bubbling fluidized bed. The aspect ratio of the bubbling fluidized bed was changed and the effects of the aspect ratio on the segregation characteristics were investigated. The segregation characteristics of a mixture of biochar and sand particles were analyzed in terms of bubble size distribution, pressure fluctuations, and mixing index. As the aspect ratio increased, the bubble size decreased, leading to a clearer segregation of biochar and sand particles. The mixing index of the biochar and sand particles decreased as the aspect ratio increased.

## Introduction

Pyrolysis is a promising thermochemical conversion technology for biomass utilization and conversion^1^. Generally, from the pyrolysis of biomass three major products are obtained such as bio-oil, biochar, and gases^2^. The yields of these products depend on different pyrolysis conditions by varying process parameters and feedstock characteristics^1–4^. Especially, The product yields of fast pyrolysis are generally 10–25, 50–70, and 10–30 wt.% for biochar, bio-oil, and gas, respectively^1^. Fast pyrolysis processes enhance bio-oil production. Bio-oil can substitute fuel oil or diesel for use in boilers, furnaces, engines, and turbines^3^. Biochar and non-condensable gas are by-products of fast pyrolysis and can be combusted to generate heat for the pyrolysis process. However, biochar can also be developed as a high-value product to improve the economic viability of fast pyrolysis processes. Biochar has received considerable attention in the environmental engineering field because of its excellent physical properties (e.g., large specific surface area^5^, porous structure^6^, and rich functional surface groups^7,8^). Many researchers have studied the benefits of biochar utilization, for example, mitigating global warming, amending soil, enhancing crop yields, carbon storage and iron/steel industry^9–14^). Furthermore, several studies have been conducted on the adsorption capability of biochar for various contaminants^15^.

Most biochars are produced via slow pyrolysis, as has been reported by multiple researchers^16,17^. These processes are generally performed in fixed-bed reactors^18^. Fixed beds have a lower efficiency than fluidized beds in terms of mass and heat transfer^19^. Fluidized beds are often used as reactors for fast pyrolysis because of their high heat and mass transfer rates for biomass and bed particles. When biomass is pyrolyzed using a bubbling fluidized bed reactor, the non-condensable gas and fine biochar are discharged from the top of the reactor, while larger biochar remains in the reactor. Biochar acts as a vapor cracking catalyst^20^. Excess biochar can crack vapor and decrease the bio-oil yield by approximately 20%. Therefore, the effective and rapid removal of biochar from bubbling fluidized bed reactors is essential. Accumulated biochar affects the pyrolysis reaction, yield, and quality of products, and the efficiency of fluidization. In particular, a constant continuous operation cannot be achieved in a large-scale reactor without periodically removal of biochar because the internal operating characteristics of the reactor based on the bed material can be changed by the accumulated char^21^. Therefore, low levels of biochar must be maintained in fluidized beds to achieve optimal fluidization and process performance. The most practical recovery process for relatively coarse biochar particles is the use of segregation coupled with an efficient removal system. In a fluidized bed, the segregation pattern of a binary mixture is a critical factor that influences heat and mass transfer, bed expansion, and process parameters. Consequently, a comprehensive understanding of the segregation dynamics of biochar is a prerequisite for optimal bed fluidization and commercial biochar production.

Several substantial literatures exist on the mixing of dissimilar binary mixtures of solids and their segregation in bubbling fluidized bed. Park and Choi^22^ investigated the segregation characteristics of biochar under differently shaped fluidized bed columns and superficial gas velocities. They then proposed the optimal superficial gas velocity and shape of a fluidized bed column to segregate biochar. Adegboye^23^ conducted experimental investigations to understand the yield and separation efficiency of recovered biochar and the effects of geometrical and operating parameters in a bubbling fluidized bed. Köhler et al.^24^ used single tracer particles and adopted Magnetic particle tracking(MPT) system to investigate the fuel mixing characteristic in a bubbling fluidized bed. Sharma et al.^25^ performed a computational fluid dynamics simulation to examine the mixing of biomass and biochar particles and the hydrodynamics of segregation in a bubbling fluidized bed. Also, Liu et al.^26^ conducted a simulation to study the detailed hydrodynamics of a thin rectangular fluidized bed at increasing pressure. Their studies investigated the effects of the particle density, size, and superficial gas velocity on the mixing and segregation behaviors of biomass and biochar. Comprehensive study of the segregation of biochar and the related gas–solid flow behavior are required to optimize the design and operating condition of a bubbling fluidized bed for fast pyrolysis. However, it is very difficult to conduct comprehensive experiments to scrutinize the segregation of biochar and the related three-dimensional gas–solid flow behavior in a fluidized bed. For reference, this comes from the limitations of experimental approaches due to complexity of multi-phase flow.

For this, this study conducted various numerical simulations of biochar segregation in a bubbling fluidized bed. The mixing and segregation of biochar were investigated by analyzing the bubble size distribution, pressure fluctuation and mixing index depending on the aspect ratio of the fluidized bed column. Finally, this study suggested fully understanding of mixing and segregation phenomena with respect to column aspect ratio in the bubbling fluidized bed. Also, if the biochar is not removed, that was piled up in a bubbling fluidized bed and finally it leads to bad quality of bio- oil and eventually the process shutdown. Hence, this research suggested the optimal design factor of the pyrolyzer especially for the quick removal of biochar.

## Methods

In this study, a numerical computational particle fluid dynamics (CPFD) scheme was used to investigate the segregation characteristics of biochar in fluidized bed columns of different shapes. The CPFD numerical scheme is based on the Eulerian–Lagrangian approach for gas–solid multiphase flows. Table 1 lists the detailed mathematical models of the gas–solid multiphase flow used in the CPFD method; a detailed description can be found in a previous study^27^. The particle–particle interactions were calculated using the particle normal stress model proposed by Harris and Crighton^28^. The Gidaspow model^29^ was used to calculate the drag function of particles.

**Table 1:** Mathematical models for the gas–solid multiphase flow in the CPFD method.

**1. Fluid phase**	
Continuity equation	
$$\frac{{\partial \left( {\varepsilon_{g} \rho_{g} } \right)}}{\partial t} + \nabla \cdot \left( {\varepsilon_{g} \rho_{g} {\mathbf{u}}_{g} } \right) = 0$$	(1)
Momentum equation	
$$\frac{{\partial \left( {\varepsilon_{g} \rho_{g} {\mathbf{u}}_{g} } \right)}}{\partial t} + \nabla \cdot \left( {\varepsilon_{g} \rho_{g} {\mathbf{u}}_{g} {\mathbf{u}}_{g} } \right) = - \nabla p - {\mathbf{F}} + \varepsilon_{g} \rho_{g} {\mathbf{g}} + \nabla \cdot \left( {\varepsilon_{g} \tau_{g} } \right)$$	(2)
Particle–fluid interaction	
$${\mathbf{F}} = \iiint {\varphi V_{p} \rho_{p} }\left[ {D_{p} \left( {{\mathbf{u}}_{g} - {\mathbf{u}}_{p} } \right) - \frac{1}{{\rho_{p} }}\nabla p} \right]dV_{p} d\rho_{p} du_{p}$$	(3)
**2. Solid phase**	
Particle acceleration equation	
$$\frac{{d{\mathbf{u}}_{p} }}{dt} = D_{p} \left( {{\mathbf{u}}_{g} - {\mathbf{u}}_{p} } \right) + {\mathbf{g}} - \frac{1}{{\rho_{p} }}\nabla p - \frac{1}{{\varepsilon_{p} \rho_{p} }}\nabla \tau_{p} + \frac{{\overline{{{\mathbf{u}}_{p} }} - {\mathbf{u}}_{p} }}{{\tau_{D} }}$$	(4)
Particle normal stress equation^25^	
$$\tau_{p} = \frac{{P_{s} \varepsilon_{p}^{\gamma } }}{{\max \left[ {\varepsilon_{cp} - \varepsilon_{p} ,\theta \left( {1 - \varepsilon_{p} } \right)} \right]}}$$	(5)
**3. Drag model**	
Gidaspow drag model^29^	
$$D_{p} = \left\{ \begin{gathered} D_{1} \quad \varepsilon_{{\text{p}}} < 0.75 \, \varepsilon_{{{\text{CP}}}} \hfill \\ \left( {D_{2} - D_{1} } \right)\left( {\frac{{\varepsilon_{p} - 0.75 \varepsilon_{CP} }}{{0.85 \, \varepsilon_{CP} - 0.75 \, \varepsilon_{CP} }}} \right) + D_{1} { 0}{\text{.75 }} \quad \varepsilon_{{{\text{CP}}}} \ge \varepsilon_{{\text{p}}} \ge 0.85 \, \varepsilon_{{{\text{CP}}}} \hfill \\ D_{2} \quad \varepsilon_{{\text{p}}} > 0.85 \, \varepsilon_{{{\text{CP}}}} \hfill \\ \end{gathered} \right.$$	(6)
Wen and Yu	
$$D_{1} = \frac{3}{8}C_{d} \frac{{\rho_{g} }}{{\rho_{p} }}\frac{{\left| {{\mathbf{u}}_{g} - {\mathbf{u}}_{p} } \right|}}{{r_{p} }}$$	(7)
$$C_{d} = \left\{ \begin{gathered} \frac{24}{{\text{Re}}}\varepsilon_{g}^{ - 2.65} \quad {\text{ Re}} < {0}{\text{.5}} \hfill \\ \frac{24}{{\text{Re}}}\varepsilon_{g}^{ - 2.65} \left( {1 + 0.15{\text{Re}}^{0.687} } \right) \quad 0.5 \le {\text{Re}} \le 1000 \hfill \\ 0.{44 }\varepsilon_{g}^{ - 2.65} \quad {\text{ Re}} > {1000} \hfill \\ \end{gathered} \right.$$	(8)
$${\text{Re}} = \frac{{2\rho_{g} r_{p} \left| {{\mathbf{u}}_{g} - {\mathbf{u}}_{p} } \right|}}{{\mu_{g} }}$$	(9)
Ergun	
$D_{2} = 0.5\left( {\frac{{C_{1} \varepsilon_{p} }}{{\varepsilon_{g} {\text{Re}} }} + C_{2} } \right)\frac{{\rho_{g} \left| {{\mathbf{u}}_{g} - {\mathbf{u}}_{p} } \right|}}{{r_{p} \rho_{p} }}$, where $C_{1} = 180$,$C_{2} = 2$	(10)

**Figure 1:**
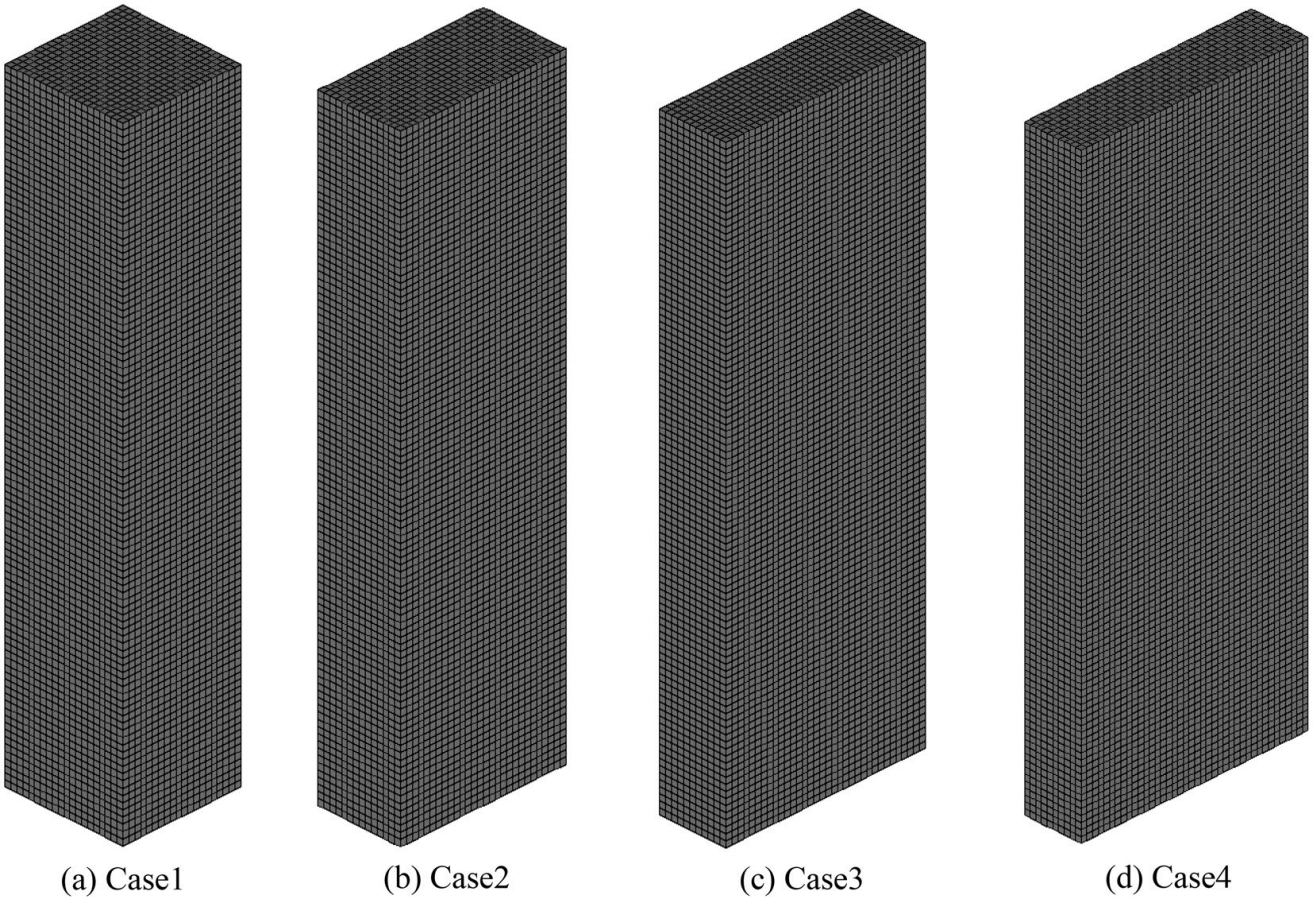
Computational domain and grids.

**Figure 2:**
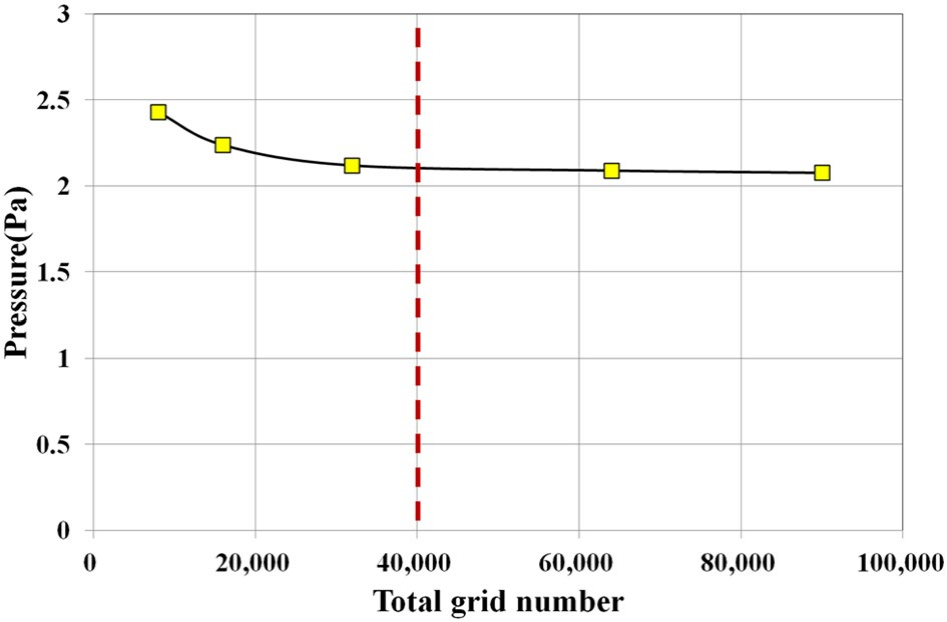
Results of the grid dependence test.

**Figure 3:**
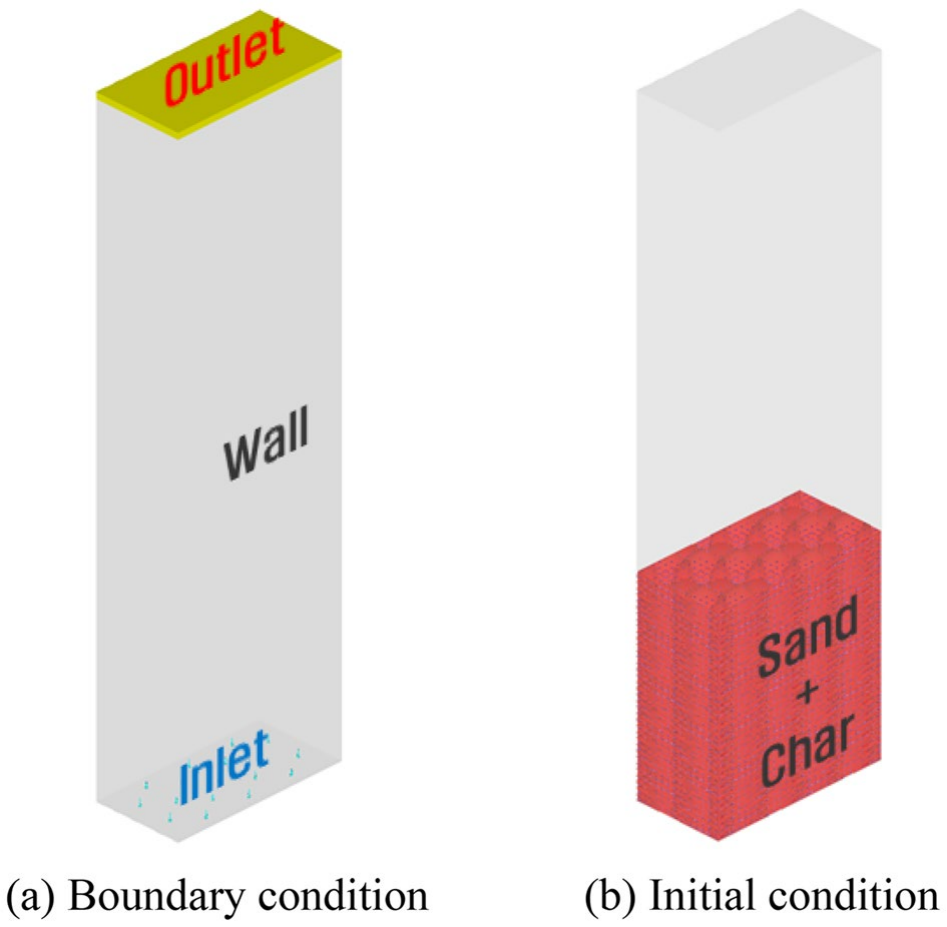
Boundary and initial conditions.

Figure 1 shows the computational domain and grids used in this study. The computational domain used four bed column shapes with different aspect ratios, and the influence of the aspect ratio on the mixing and segregation of biochar was analyzed. The dimensions of the computational domain are listed in Table 2. The aspect ratios in Cases 1, 2, 3, and 4 were 1:1, 2:1, 3:1, and 4:1, respectively. The cross-sectional area of the fluidized bed column was 0.01 m^2^ for all fluidized bed columns. Figure 2 shows the results of the grid dependence test conducted prior to the main calculation and this pressure is the time-averaged value at the bed height of 10 mm. After the grid number of 40,000, the average pressure does not change, hence the gird number was selected. A computational grid was generated using a hexahedral mesh. The boundary and initial conditions for the computational domain are presented in Fig. 3 and Table 3, respectively. A constant-velocity boundary condition was applied at the inlet of the computational domain. The superficial gas velocity was maintained at a constant value for all computational domains to focus solely on the influence of the aspect ratio. The superficial gas velocity was fixed at U/U_mf_ = 1.35 as per Park and Choi’s experiment^22^ for sand and biochar mixing. Here, U_mf_ represents the superficial gas velocity at minimum fluidization. In addition, the superficial gas velocity of U/U_mf_ = 1.35 ensures a residence time of 2 s for the pyrolysis gas in the fast pyrolyzer^1–3^. This condition is extremely important for ensuring a high bio-oil yield and quality from the fast pyrolysis of biomass. A constant-pressure boundary condition was used at the outlet of the fluidized bed column. The particles and gas were assumed to experience partial slip and no slip, respectively, at the wall boundaries. In the fluidized bed column, sand and biochar were initially added to the fluidized bed column and fluidized by air. The sand was considered as jetsam and the biochar was defined as flotsam to investigate their mixing and segregation. The material properties of biochar and sand are listed in Table 4. A computational analysis was conducted using BARRACUDA ver.16.0.3 CPFD analysis code.

**Table 2:** Dimensions of the computational domain.

Case	Aspect ratio (length:width)	Cross-sectional area (m^2^)	Height (m)
1	1:1	0.01	0.5
2	2:1		
3	3:1		
4	4:1

**Table 3:** Boundary conditions.

Boundary	Conditions
Inlet	Constant velocity (U/U_mf_ = 1.35)
Outlet	Constant pressure 101,325 Pa (atmospheric)
Wall	Partial slip of particles No slip of gas

**Table 4:** Properties of particles.

Particles	Mean diameter^22^ ($\upmu$m)	Particle void fraction (1−${\upvarepsilon }_{p}$)	Bulk density^22^ (kg/m^3^)	Geldart classification
Sand	387	0.33	1,590	B
Char	957	0.69	120	A

## Results and discussion

### Validation of computational procedure

**Figure 4:**
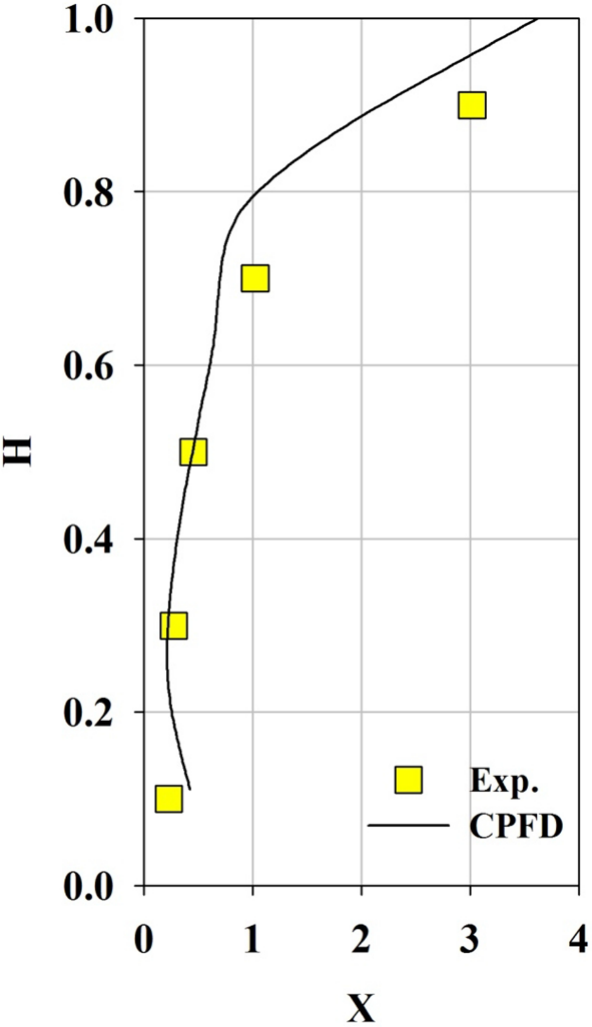
Comparison between the CPFD analysis and experimental data.

**Figure 5:**
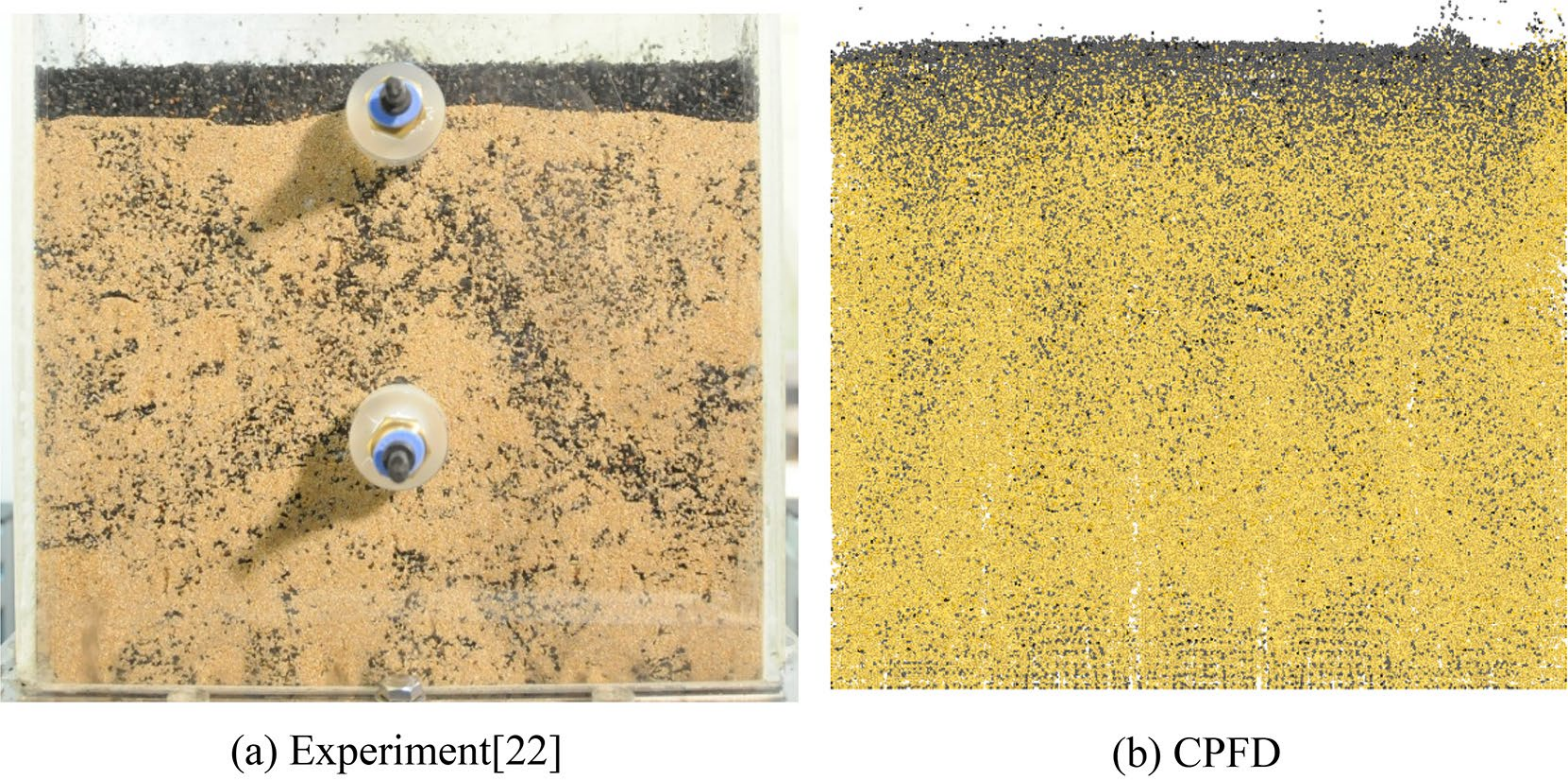
Behavior of char particle in a bubbling fluidized bed at U/Umf = 1.35.

To validate the CPFD numerical scheme, the mixing and segregation of biochar in a rectangular bubbling fluidized bed were calculated under the same conditions as those used by Park and Choi^22^. Figure 4 shows a comparison of the results of the CPFD simulation and experiment and indicates the mass fraction of biochar in the fluidized bed column at U/Umf = 1.35. In the figure, X is the local mass fraction of biochar divided by the average biochar mass fraction and H represents the dimensionless bed height. H was divided by the total bed height. As shown in Fig. 4, the results of the CPFD numerical scheme agreed with the experimental results, although there were minor differences near the top of the bed. Figure 5a,b show snapshots of the segregation of biochar particles in the fluidized bed obtained from the experiment and CPFD simulation, respectively. The biochar floated to the top of the bed in both the experiment and the CPFD simulation. Hence, the mixing and segregation behavior of biochar in the CPFD numerical scheme was similar to that in the experimental results. Thus, the computational procedure adopted in this study can be applied with reasonable accuracy for future segregation simulations.

### Hydrodynamic characteristics with respect to bed aspect ratio

**Figure 6:**
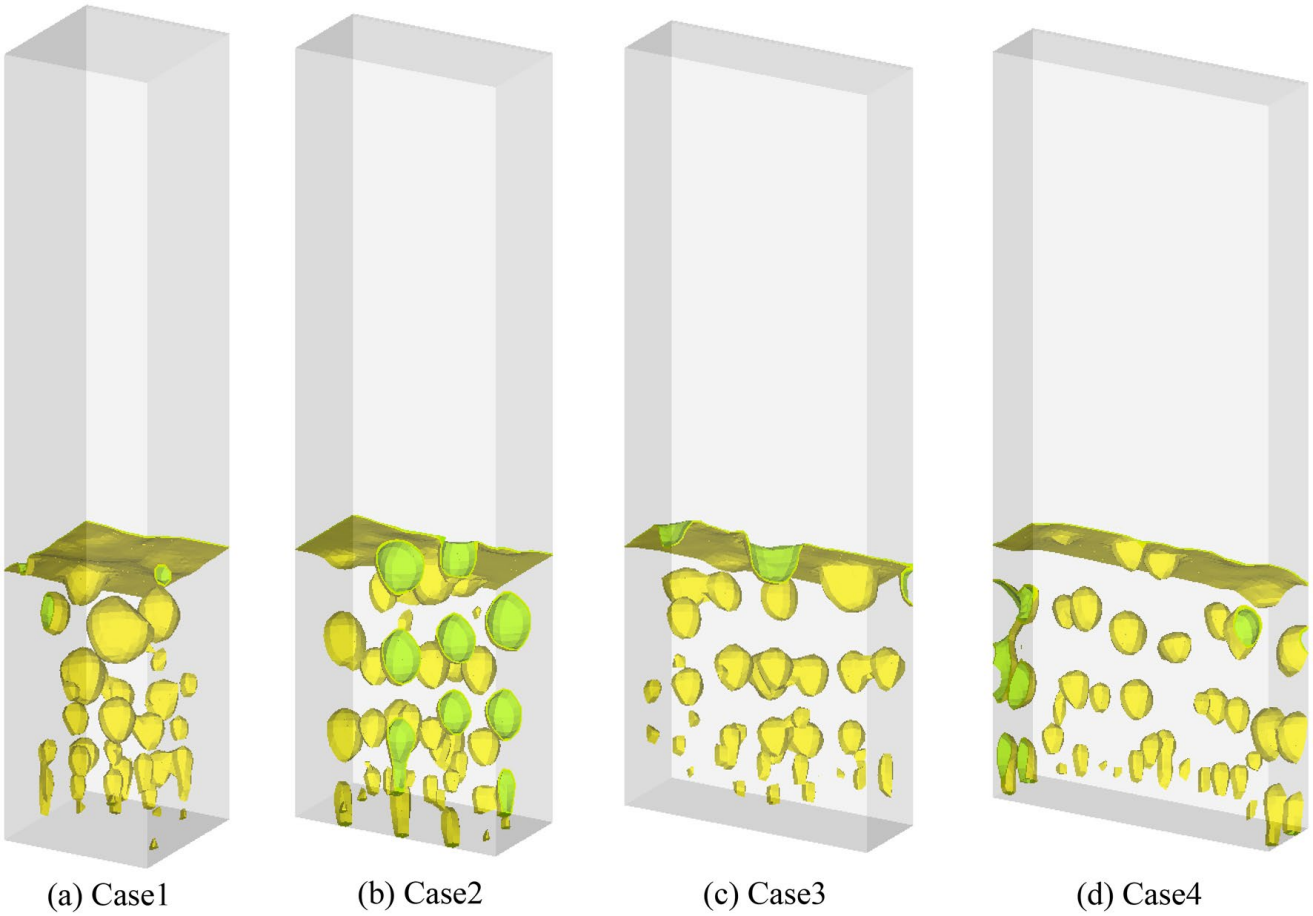
Bubbles for different shapes of fluidized bed column.

**Figure 7:**
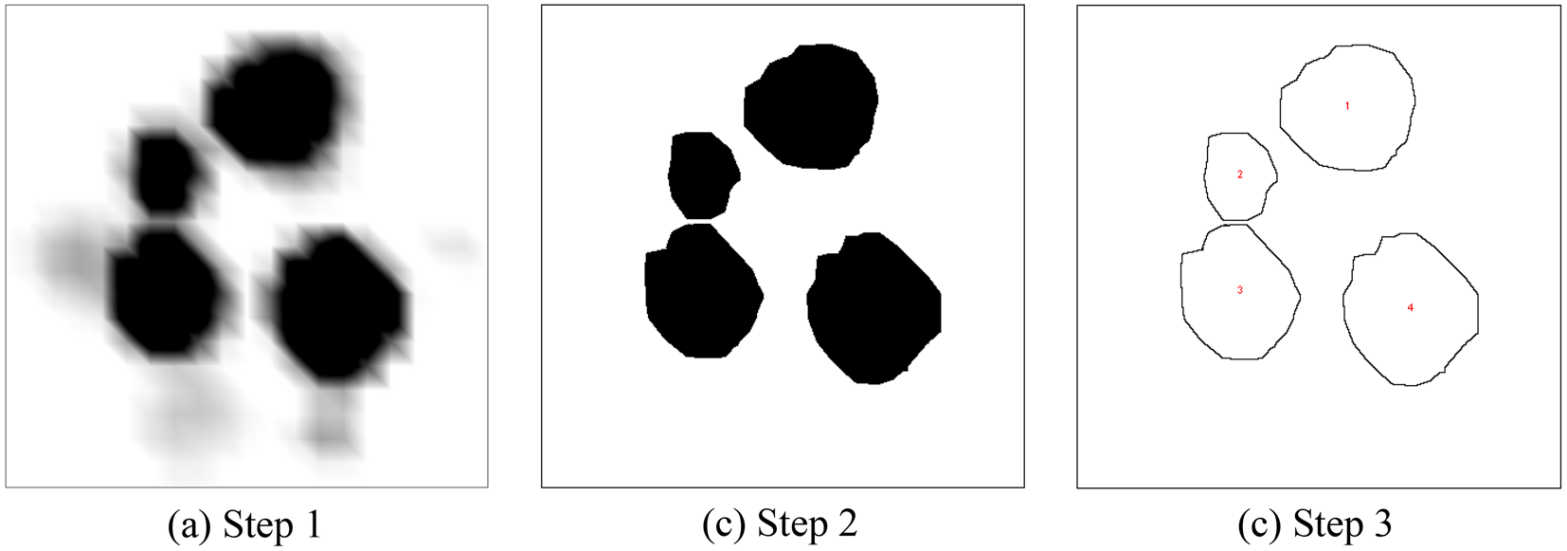
Image processing procedure for calculation of bubble diameter.

Figure 6 shows the isosurface of the instantaneous gas volume fraction in the bubbling fluidized bed column. The threshold value of the gas volume fraction defined the bubble dimensions; previous studies often employed different threshold values^30–32^. This study selected 0.55 as the threshold value to perform the phase identification. The details of image processing method can be found in a previous study^27^. The formation of bubbles near the bed bottom, as well as their growth, coalescence, and splitting, are shown in Fig. 6. An image analysis technique was used to obtain equivalent bubble diameters and the procedure is illustrated in Fig. 7. Black and white represent the bubble and emulsion phases, respectively. The captured images were converted to grayscale (Fig. 7a) and then converted to a binary image (Fig. 7b). Subsequently, bubble edges were detected (Fig. 7c). Finally, the bubble area was calculated using the number of enveloped pixels in the detected edge and a scaling factor based on the bed width. The diameter of a bubble was calculated from the area equivalent to Eq. (1) ^33^.

1 $${\mathrm{A}}_{\mathrm{b}}{\mathrm{D}}_{\mathrm{b}}=\sqrt{4{\mathrm{A}}_{\mathrm{b}}/\uppi }$$

**Figure 8:**
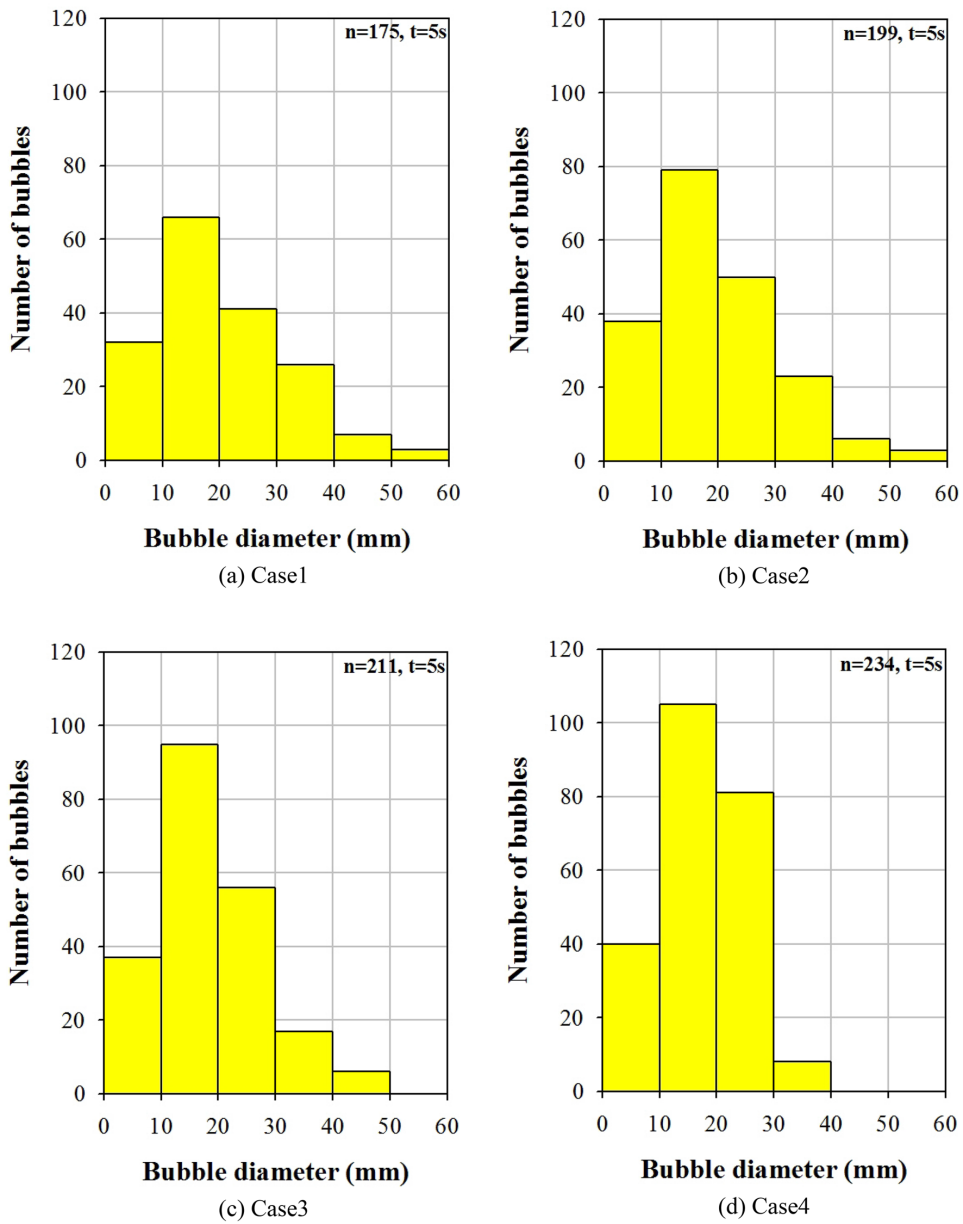
Bubble diameter distributions for different shapes of fluidized bed columns.

**Figure 9:**
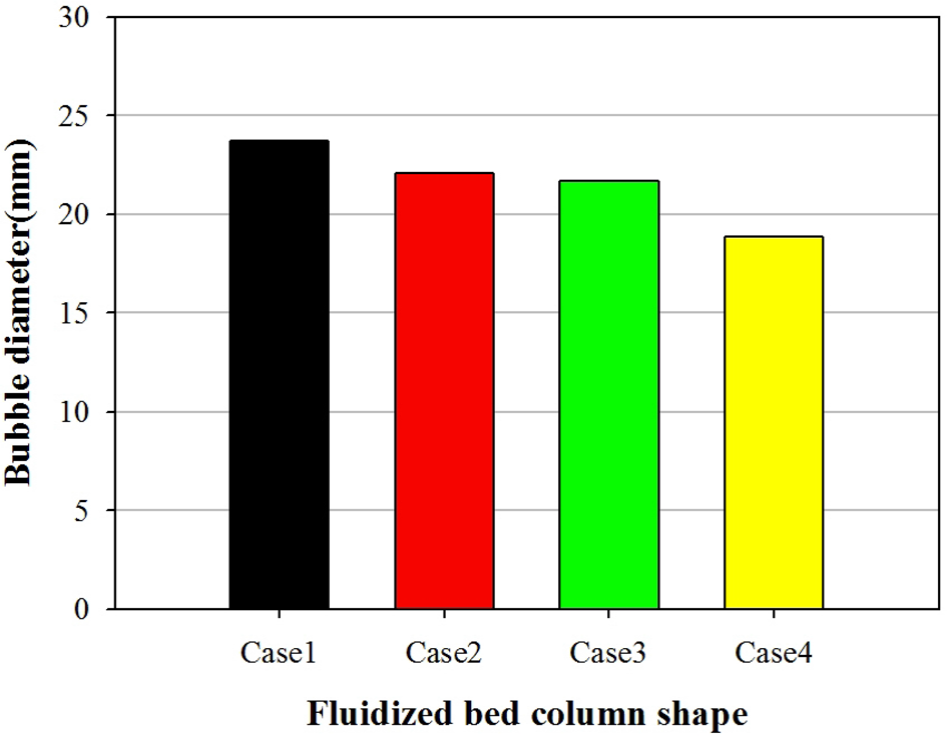
Bubble diameter for different shapes of fluidized bed columns.

Figures 8 and 9 show the distribution of the bubble diameters passing through the measured cross-section and time-averaged bubble diameter, respectively. The height of the cross section was 60 mm from the bottom of the bed. Time averaging was performed for 5 s. As shown in Fig. 8, the number of bubbles increased from 175 to 234 when the aspect ratio of the fluidized bed column increased from 1:1 to 4:1. The number of smaller bubbles with diameters in the range of 10–30 mm increased from Cases 1 to 4. However, the number of larger bubbles with diameters of 40–60 mm decreased. The relatively small bubble sizes and large number of bubbles are likely attributable to the aspect ratio of the fluidized bed column. The same flow pattern and bubble behavior were previously observed in 2D experiments^34^. When the width of the fluidized bed was small, the growth of bubbles was prohibited by the walls and the probability of coalescence between neighboring bubbles decreased. This pattern was observed via the distribution of the bubble diameters (Fig. 8). When the aspect ratio was 1:1, several bubbles coalesced to form larger ones (≥ 40 mm). Conversely, when the aspect ratio was 4:1, only a few bubbles coalesced to form larger ones (≥ 40 mm). Most bubbles had a diameter between 10 and 30 mm. As shown in Fig. 9, the averaged bubble diameter decreased from 23.7 to 18.9 mm when the aspect ratio increased from 1:1 to 4:1.

**Figure 10:**
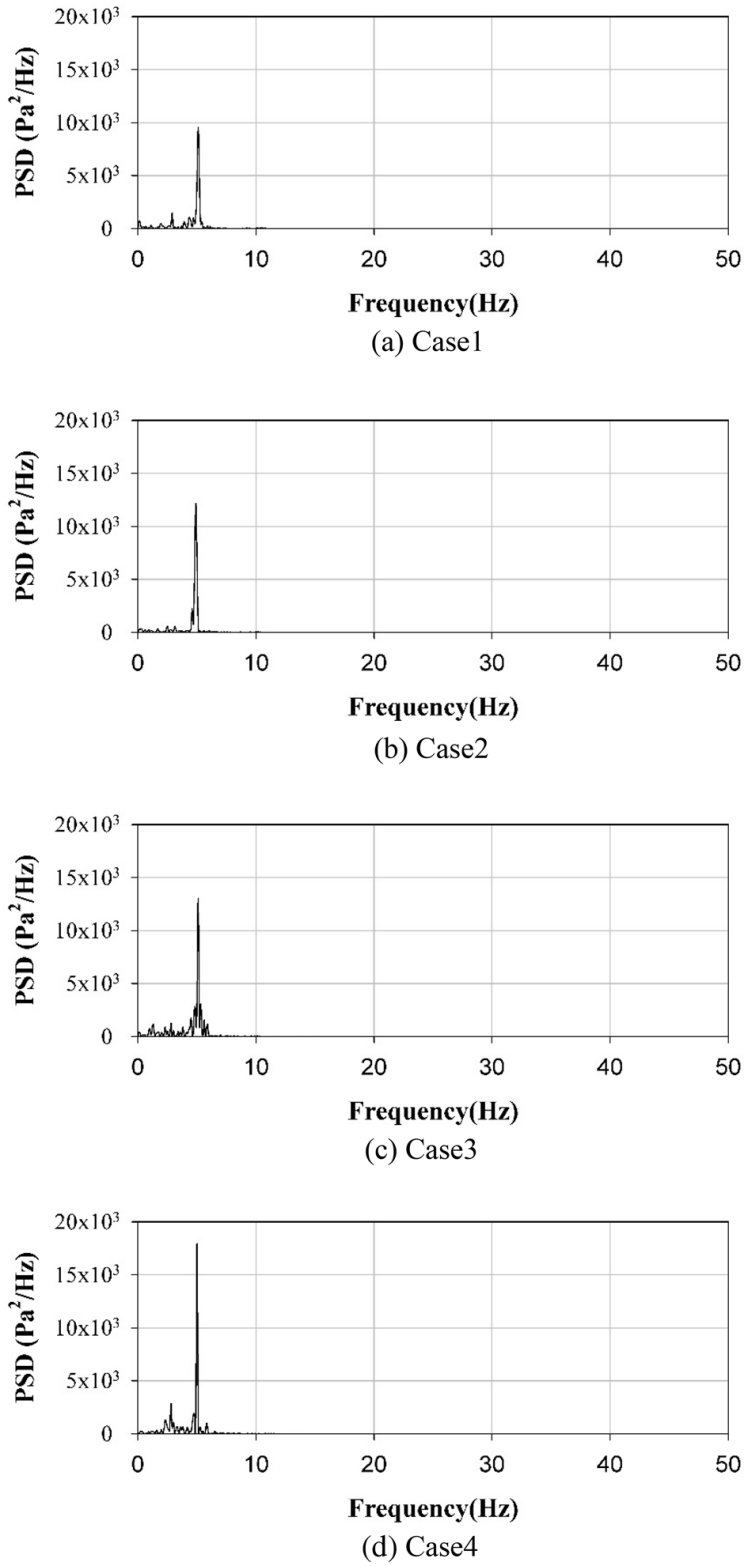
Power spectral density of pressure fluctuation for different shapes of fluidized bed columns.

Many researchers have investigated pressure fluctuation signals in fluidized beds to obtain information on gas–solid dynamics. Their focus has mainly been on decoupling the pressure fluctuation signal to obtain useful information. The interpretation of pressure fluctuation signals is typically performed using analyses of chaos or state-space, frequency or correlation, and time. In this study, we calculated the power spectral density (PSD) of the pressure fluctuation signal to examine the effect of bubble size on the hydrodynamic characteristics of a bubbling fluidized bed. The PSD with respect to the aspect ratio of the fluidized bed was calculated using MATLAB software, as shown in Fig. 10. Further details about the PSD can be found in the previous work^27^. The frequency spectrum of the pressure fluctuation exhibited multiple peaks for all cases, and the dominant frequencies were between 4.8 and 5.2 Hz. This frequency spectrum indicates that the flow regime represents multiple-bubbling fluidization. The PSD results of this study were in accordance with those of previous studies^35,36^. As shown in Fig. 9, most generated bubbles were between 10 and 30 mm in size and the proportion of small bubbles increased as the aspect ratio increased. Therefore, the dominant frequency was attributed to bubbles between 10 and 30 mm in size. When the aspect ratio increased from 1:1 to 4:1, the dominant frequency of the pressure fluctuation increased from 4.8 to 5.2 Hz. This is because the bubble diameters decreased at higher aspect ratios. Generally, large gas bubbles generate low-frequency waves, whereas small gas bubbles generate high-frequency waves. Notably, when the aspect ratio was 4:1, the dominant frequency of the pressure fluctuation was the highest among the four cases. These bubble characteristics influenced the segregation of biochar, as described in the following subsection.

### Segregation characteristics with respect to bed aspect ratio

**Figure 11:**
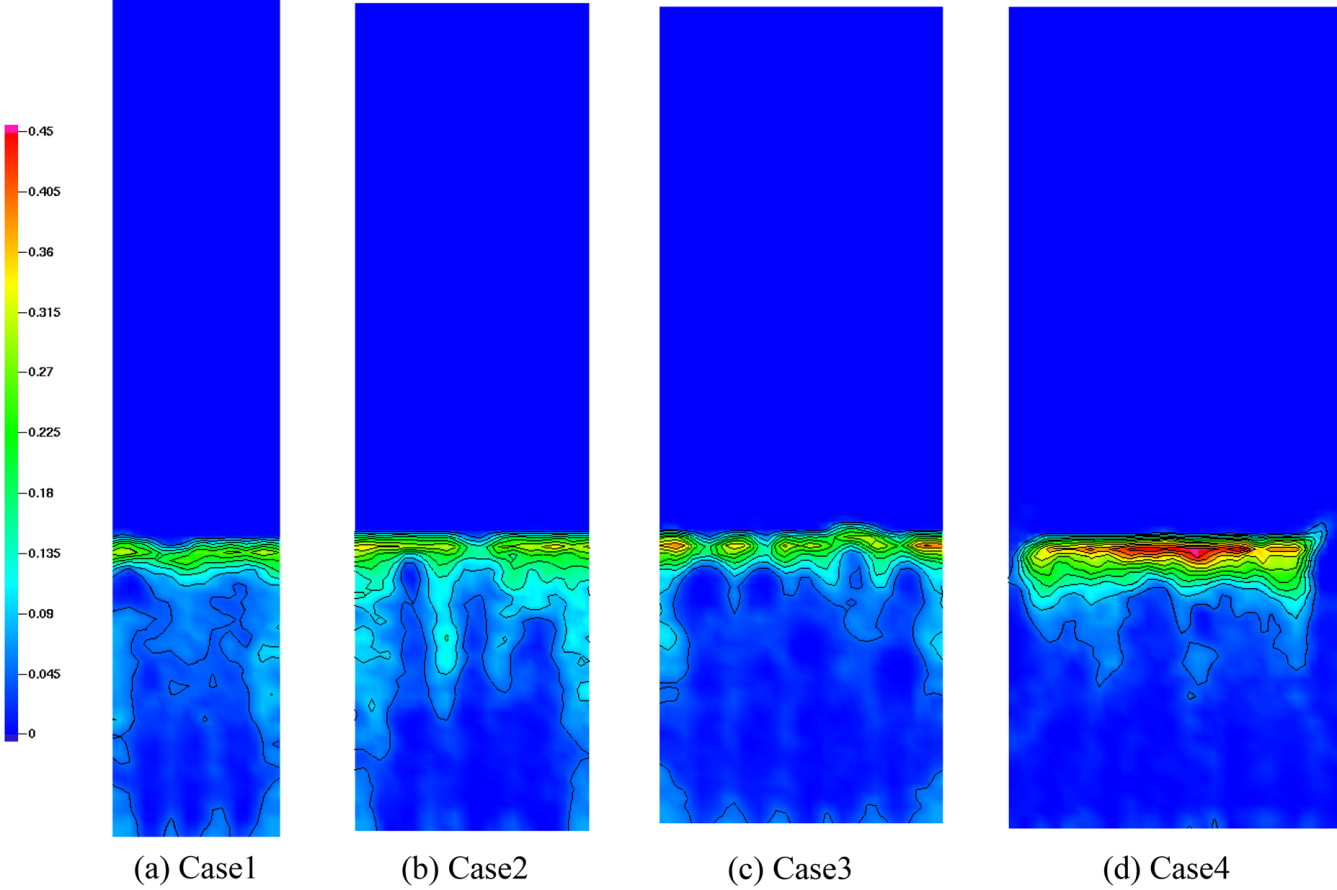
Contours of bio-char mass fraction for different shapes of fluidized bed columns.

**Figure 12:**
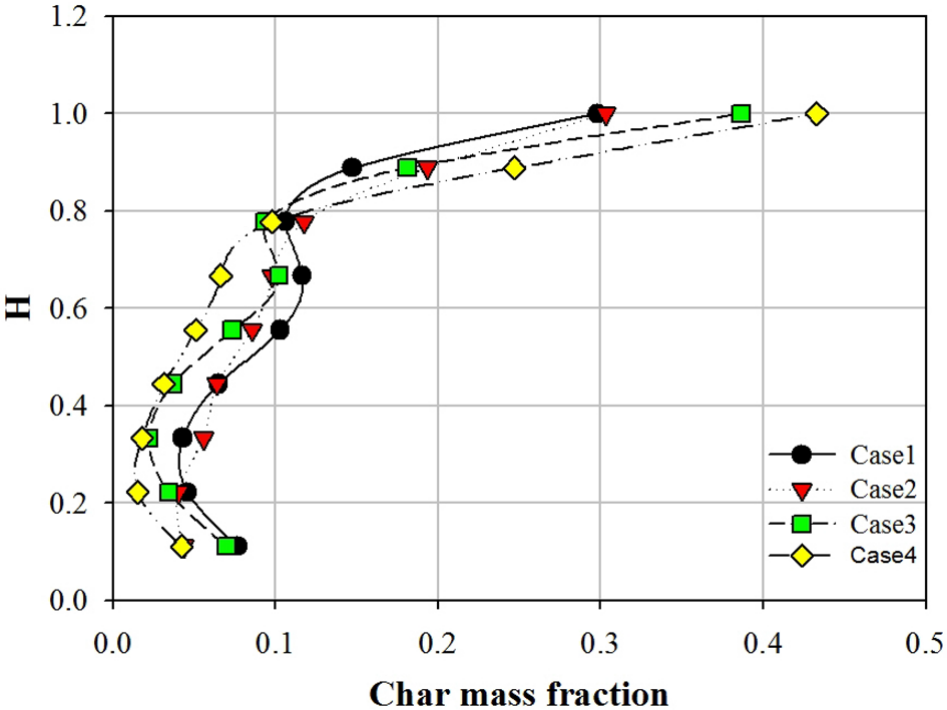
Axial bio-char mass fraction for different shapes of fluidized bed columns.

**Figure 13:**
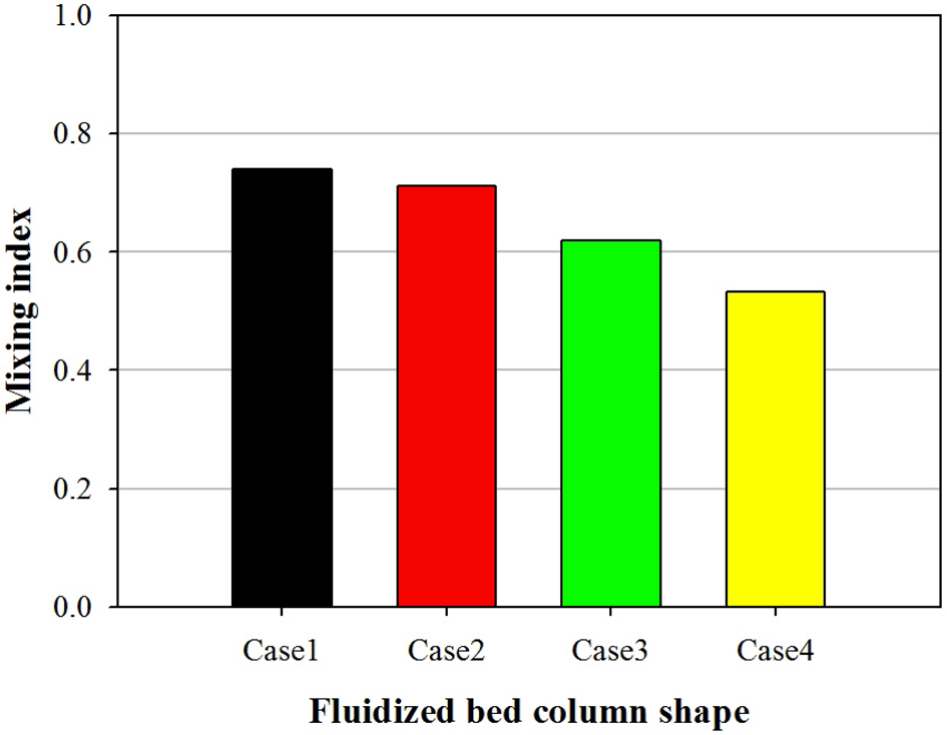
Mixing index for different shapes of fluidized bed columns.

The mixing and segregation of a binary mixture in fluidized beds are mainly determined by the superficial gas velocity. Nevertheless, other parameters, such as particle size and shape, also play a role. The shape of the fluidized bed column is an important parameter that controls particle mixing and segregation^37^. To investigate this effect, four fluidized bed columns with different aspect ratios (1:1, 2:1, 3:1, and 4:1) were numerically simulated. As discussed in Sect. 3.2, bubble characteristics play an important role in particle mixing and segregation. In a gas–solid fluidized bed, particles in the vicinity of bubbles are sucked into the bubble wake and then move to the upper part of the bed with the bubbles. Eventually, the particles are distributed at the top of the bed once the bubbles collapse^38^. In addition, the fluidized bed contracts and expands periodically as the bubbles rise to the top of the bed. Consequently, high-density sand moves to the bottom of the bed, whereas low-density biochar moves to the top of the bed. This phenomenon increases the mass fraction of biochar at the top of the fluidized bed and decreases it at the bottom. Figure 11 shows the contours of the mass fraction of biochar in the fluidized bed with respect to the aspect ratio. The mass fraction of biochar is high in the upper part of the bed and low in the lower part. Figure 12 shows the distribution of the biochar mass fraction in accordance with the aspect ratio along the axial direction. In Fig. 12, H is the bed height, which is nondimensionalized by the total bed height. When the aspect ratio increased from 1:1 to 4:1, the biochar mass fraction at the upper part (H = 1) of the fluidized bed increased from 0.29 to 0.43, whereas that at the lower part (H = 0.1) decreased from 0.07 to 0.04. These phenomena are affected by the bubble characteristics, particularly bubble size and movement. The mass fraction of biochar at the top of the bed increased as the bubble diameter decreased. Figure 13 shows the mixing indices for the four fluidized bed column shapes. Kramer’s mixing index, as shown in Eq. (2), was used to calculate the segregation of the biochar^39^. In Eq. (2), *X*_*i*_ represents the mass fraction of char in each sampling cell, and ${\sigma }_{0}$ is the standard deviation of the mass fraction of char when the sand and char were completely segregated. ${\sigma }_{r}$ is the standard deviation of the mass fraction of char when the sand and char were completely mixed.

2 $$M=\frac{{\sigma }_{0}-\sigma }{{\sigma }_{0}-{\sigma }_{r}}, \sigma =\sqrt{\frac{1}{n-1}\sum_{i=1}^{n}{\left({X}_{i}-\overline{X }\right)}^{2}}$$

A mixing index of 1 indicates complete mixing, whereas a mixing index of 0 indicates complete segregation. The mixing index of sand and biochar decreased from 0.73 to 0.53 as the aspect ratio increased from 1:1 to 4:1. As per these results, an aspect ratio of 4:1 was the most appropriate for separating biochar from the bubbling fluidized bed.

## Conclusions

In this numerical study, the segregation characteristics of biochar in a bubbling fluidized bed were investigated by varying the aspect ratio of the fluidized bed column. As the aspect ratio of the fluidized bed column increased, the bubble size decreased. Moreover, the bubble size had a significant influence on the segregation characteristics of biochar in the bubbling fluidized bed. As the aspect ratio of the fluidized bed column increased from 1:1 to 4:1, the number of bubbles increased from 175 to 234. Additionally, the mean bubble diameter decreased from 23.7 to 18.9 mm. When the aspect ratio was 1:1, bubbles had the greatest opportunity to coalesce and ultimately grow. Consequently, bubbles larger than or equal to 40 mm formed. However, when the aspect ratio was 4:1, only a few bubbles coalesced. Under these circumstances, small bubbles, with sizes between 10 and 30 mm, were abundantly produced. The dominant frequency of the pressure fluctuation in the fluidized bed increased from 4.8 to 5.2 Hz as the aspect ratio changed from 1:1 to 4:1. In addition, the magnitude of the PSD increased. When large bubbles were present, the dominant frequency of pressure fluctuations was small. This bubble behavior significantly influenced the segregation between the biochar and sand. The biochar mass fraction in the upper part (H = 1) of the fluidized bed increased from 0.29 to 0.43 as the aspect ratio increased from 1:1 to 4:1. Consequently, the mixing index of sand and biochar decreased from 0.73 to 0.53. The biochar segregated the quickest when the aspect ratio of the fluidized bed column was high. Therefore, a fluidized bed column with a high aspect ratio is efficient for segregating the biochar produced during the fast pyrolysis process.

## Data Availability

The datasets used in the current study are available from the corresponding author upon reasonable request.

## Abbreviations

$$C_{d}$$: Drag coefficient
$$D_{p}$$: Drag function
${\mathbf{F}}$: Rate of momentum exchange per volume between the fluid and particle phases (N/m^3^s)
$${\mathbf{g}}$$: Gravitational acceleration (m^2^/s)
$p$: Fluid pressure (Pa)
$P_{s}$: Pressure constant of the solid-phase stress model (Pa)
$${\text{Re}}$$: Reynolds number
$$r_{p}$$: Particle radius (m)
$${\mathbf{u}}_{g}$$: Fluid velocity (m/s)
$${\mathbf{u}}_{p}$$: Particle velocity (m/s)
$$V_{p}$$: Particle volume (m^3^/s)
X: Local mass fraction of biochar divided by the average biochar mass fraction
H: Dimensionless bed height

## Greek letters

$\gamma$: Dimensionless constant of the solid-phase stress model
$\varepsilon_{cp}$: Closed pack particle volume fraction
$\varepsilon_{g}$: Fluid volume fraction
$\varepsilon_{p}$: Particle volume fraction
$\theta$: Dimensionless constant of the solid-phase stress model
$\mu_{g}$: Fluid viscosity (Pa s)
$\rho_{g}$: Fluid density (kg/m^3^)
$\rho_{p}$: Particle density (kg/m^3^)
$\tau_{D}$: Collision damping time (s)
$\tau_{g}$: Fluid stress tensor (Pa)
$\tau_{p}$: Particle normal stress (Pa)
